# Association of pronounced elevation of NET formation and nucleosome biomarkers with mortality in patients with septic shock

**DOI:** 10.1186/s13613-023-01204-y

**Published:** 2023-10-17

**Authors:** Muzhda Haem Rahimi, Frank Bidar, Anne-Claire Lukaszewicz, Lorna Garnier, Léa Payen-Gay, Fabienne Venet, Guillaume Monneret

**Affiliations:** 1 Hospices Civils de Lyon, Guillaume Monneret - Immunology Laboratory, Hôpital E. Herriot, Lyon, France; 2 Université de Lyon, EA 7426 “Pathophysiology of Injury-Induced Immunosuppression”, Université Claude Bernard Lyon_1, Lyon, France; 3 Hospices Civils de Lyon, Anesthesiology and Critical Care Medicine Department, Hôpital E. Herriot, Lyon, France; 4 Hospices Civils de Lyon, Immunology Laboratory, CH Lyon-Sud, Lyon, France; 5 Center for Innovation in Cancerology of Lyon (CICLY) EA 3738, Faculty of Medicine and Maieutic Lyon Sud, Claude Bernard University Lyon I, 69921 Oullins, France; 6 NLRP3 Inflammation and Immune Response to Sepsis Team, Centre International de Recherche in Infectiology (CIRI), Inserm U1111, CNRS, UMR5308, Ecole Normale Supérieure de Lyon, Claude Bernard University Lyon 1, Lyon, France

**Keywords:** Sepsis, Septic shock, Inflammation, NETs, NETosis, Nucleosome, Immunosuppression, mHLA-DR

## Abstract

**Background:**

Understanding the mechanisms underlying immune dysregulation in sepsis is a major challenge in developing more individualized therapy, as early and persistent inflammation, as well as immunosuppression, play a significant role in pathophysiology. As part of the antimicrobial response, neutrophils can release extracellular traps (NETs) which neutralize and kill microorganisms. However, excessive NETs formation may also contribute to pathogenesis, tissue damage and organ dysfunction. Recently, a novel automated assay has been proposed for the routine measurement of nucleosomes H3.1 (fundamental units of chromatin) that are released during NETs formation. The aim of the present study was to measure nucleosome levels in 151 septic shock patients (according to sepsis-3 definition) and to determine association with mortality.

**Results:**

The nucleosome H3.1 levels (as determined by a chemiluminescence immunoassay performed on an automated immunoanalyzer system) were markedly and significantly elevated at all-time points in septic shock patients compared to the control group. Immunological parameters indicated tremendous early inflammation (IL-6 = 1335 pg/mL at day 1–2) along with marked immunosuppression (e.g., mHLA-DR = 3853 AB/C and CD4 = 338 cell /µL at day 3–4). We found significantly positive correlation between nucleosome levels and organ failure and severity scores, IL-6 concentrations and neutrophil count. Significantly higher values (day 1–2 and 3–4) were measured in non-survivor patients (28-day mortality). This association was still significant after multivariate analysis and was more pronounced with highest concentration. Early (day 1–2) increased nucleosome levels were also independently associated with 5-day mortality. At day 6–8, persistent elevated nucleosome levels were negatively correlated to mHLA-DR values.

**Conclusions:**

This study reports a significant elevation of nucleosome in patients during a one-week follow-up. The nucleosome levels showed correlation with neutrophil count, IL-6 and were found to be independently associated with mortality assessed at day 5 or 28. Therefore, nucleosome concentration seems to be a promising biomarker for detecting hyper-inflammatory phenotype upon a patient's admission. Additional investigations are required to evaluate the potential association between sustained elevation of nucleosome and sepsis-induced immunosuppression.

**Supplementary Information:**

The online version contains supplementary material available at 10.1186/s13613-023-01204-y.

## Introduction

Sepsis is a critical global health issue and a significant contributor to morbidity and mortality worldwide. Over years, the mortality rate of septic shock has remained high, ranging from 20 to 50%, despite advances in critical care management. A recent epidemiological study indicated that sepsis was responsible for approximately 11 million deaths globally in 2017, representing about 20% of all global deaths [1].

Sepsis and septic shock are defined as a life-threatening organ dysfunction caused by a dysregulated host response to infection [2]. Both conditions are characterized by a profound systemic inflammatory response that leads to widespread tissue damage, multiple organ failure, and often results in death. This immune-inflammatory response is at the forefront of sepsis pathophysiology. There is a hyper-inflammatory phase with a massive release of inflammatory mediators that is simultaneously accompanied by compensatory mechanisms (both anti-inflammatory and immunosuppressive) aimed at blocking excessive deleterious inflammation [3–5]. If this counter-response is too intense or cannot be resolved, it can lead to a state of acquired immunodeficiency. It is usually called sepsis-induced immunosuppression and leaves patients vulnerable to both primary (absence of clearance of initial foci) and secondary infections by opportunistic microorganisms or to viral reactivations [6–8]. Understanding the mechanisms underlying immune dysregulation in sepsis is a major challenge in developing more individualized therapy, as early and persistent inflammation, as well as immunosuppression, play a significant role in pathophysiology.

Neutrophils are the first line of defense in the innate immune response against invading pathogens. They combat invading pathogens through various mechanisms, including phagocytosis, production of reactive oxygen species; and release of chemoattractant mediators and inflammatory compounds that amplify the initial response [9–11]. In addition, neutrophils produce extracellular traps (NETs) by releasing their chromatin and granule proteins to form extracellular fibers which consist of a meshwork of DNA, histones, and antimicrobial proteins [12]. Among these components, the nucleosome stands as the fundamental unit of decondensed chromatin, consisting of DNA intricately wound around a histone core. The core histone proteins H3, H4, H2A, and H2B were identified as major constituents of NETs, constituting approximately 70% of NETs proteins [13]. Specifically, circulating histone H3 was primarily attributed to neutrophils under septic conditions. Moreover, histone H3.1, a variant of histone H3 featuring distinct post-translational modifications, has been implicated in NETs formation and correlated with its antimicrobial potency [14, 15]. Consequently, the quantification of circulating H3.1-nucleosomes has emerged as a dependable proxy for assessing NETs levels in plasma.

The DNA and histones form the backbone of NETs formation, while the antimicrobial proteins, such as neutrophil elastase, myeloperoxidase, and cathelicidin, provide a potent defense against invading pathogens [14]. NETs are thus additional weapons to immobilise, trap and kill invading organism. Although the primary role of NETs is obviously beneficial to the host, there is increasing evidence that excessive (or poorly controlled) activation leads to excess inflammation [16, 17], endothelial damage and harm [18, 19]. Indeed, the different NETS constituents can by themselves act as damage-associated molecular patterns (DAMPs) that activate the immune system and exacerbate inflammation [20]. Therefore, not surprisingly, NETs have been implicated in adverse outcomes in various conditions [21], including severe sepsis [22, 23], inflammation [24], COVID-19 [25]. Their levels have been shown to correlate with severity of illness and organ failure and to be associated with mortality [26–30]. Nucleosomes, as surrogate of NETs formation, appear therefore as both biomarkers and potential actors of deleterious inflammation.

Recently, a new automated and standardized NET assay has been developed and CE marked for determining the release of NETs measured by circulating H3.1-containing nucleosomes. In this study, we utilized this novel assay to investigate nucleosome levels in patients diagnosed with septic shock. The primary aim of this exploratory study was to evaluate the association between nucleosome levels and mortality in these patients. Additionally, we analyzed traditional markers of immunomonitoring, such as neutrophil count, IL-6, monocyte HLA-DR (mHLA-DR), and lymphocyte count, to further understand the potential role of nucleosome levels in septic shock.

## Methods and materials

### Patients

We included patients admitted to ICU in Anesthesiology and Intensive Care Department (Hôpital E. Herriot, Hospices Civils de Lyon) with septic shock. This work is a sub-study of the IMMUNOSEPSIS study registered at clinicaltrials.gov (NCT04067674). Blood samples were obtained and introduced in EDTA tubes at day (D): D1-2, D3-4 and D6-8 (one sample per period). Informed consent and non opposition to participation to this study was systematically obtained from the patient or a third party before any blood sampling was performed and was recorded in patient’s clinical file. All procedures performed were in accordance with the ethical standards of the institutional and/or national research committee (Comité de Protection des Personnes Ouest II, IRB number #19.01.23.71957) and with the 1964 Helsinki declaration and its later amendments or comparable ethical standards. Clinical and biological databases were reported to the National Commission for Information Technology and Freedom (CNIL, number 08-27). A written non-opposition to the use of donated blood for research purposes was obtained from healthy volunteers (*n* = 50). Usual clinical data of patients such as age, sex, severity scores, exposure to invasive device, primary sit of infection, nosocomial infection appearance, length of stay in ICU and mortality on day 28 were collected.

### Nu.Q H3.1® ImmunoAssay

Nucleosomes were measured using Nu.Q H3.1® Immunoassay (Belgian Volition SRL, Isnes, Belgium) according to the manufacturer’s instruction. Briefly, this sandwich immunoassay is based on magnetic beads and chemiluminescence technology and is performed on the IDS-i10 automated immunoanalyzer system (Immunodiagnostic Systems Ltd, UK). Fifty μL of K2-EDTA plasma are incubated with acridinium ester labeled anti-nucleosome antibody. Then, magnetic particle beads, coated with the monoclonal anti-histone modification capture antibody are added. Finally, after a wash step, trigger solutions are added, and the light emitted by the acridinium ester is measured by the luminometer system. The results are expressed in relative light unit (RLU) and the concentrations are extrapolated using a four-parameter logistic regression of a reference standard curve. All samples were analyzed in duplicate.

### Additional immunological parameters

The concentration of interleukin-6 (as pg/ml) was measured using Ella automated immunoassay system (Bio-Techne). Neutrophils, lymphocyte count and monocyte HLA-DR (mHLADR) expression were analysed using flow cytometer (Beckman Coulter). Results were expressed as number of cell / microL or, in case of mHLA-DR, as total number of antibody bound per cells (AB/C).

### Statistical analysis

Statistical analyses were carried out using R Studio (R version 4.2.2). We employed chi-square and Mann–Whitney tests for comparisons between qualitative and quantitative variables, respectively, while Kruskal–Wallis with Dunn's multiple comparison test was used for the evaluation of multiple groups of quantitative variables. Spearman’s rho correlation test has been used to assess correlation between parameters. Results were expressed as median ± interquartile range (IQR). The Kaplan–Meier curve was used to evaluate the effect of nucleosome H3.1 individually or in combination with interleukin-6 on survival probability. Cox proportional hazard model was used to assess the association of circulating nucleosome with mortality. Receiver operating characteristic (ROC) was constructed to explore the relationship between the sensitivity and specificity of nucleosome H3.1 for day-5 and day-28 mortality. As this is an exploratory study without a priori hypothesis, no sample size calculation has been performed.

## Results

Overall, 151 patients were included representing 345 samples as follows: 77 patients had 3 samples (D1-2, 3–4, 6–8), 40 patients had 2 samples (D1-2, 3–4), and 34 patients had a single sample (D1-2). Table 1 depicts the main demographic and clinical characteristics of patients according to 28 day-mortality. Median age was 69 and, globally, it was a severe septic shock cohort (mortality = 45%, SAPS II = 65 [54–79], SOFA = 10 [8–13]). In agreement, patients presented with marked inflammation, IL-6 = 1335 pg/mL [338–3871] at Day 1–2 and profound cellular alterations, e.g., at Day 3–4, mHLA-DR = 3853 AB/C [2536–6919] and CD4 = 338 cells/µL [221–540]. Comprehensive results concerning immunomonitoring can be found in Additional file 2: Table S1.

**Table 1:** Main patients’ characteristics according to 28-day mortality

	All patients (151)	Non-survivors (68)	Survivors (83)	*p*
Gender, *n* (%)	98 (64.9)	47 (69.11)	51 (61.45)	0.42
Age_median (IQR)	69 (62–80)	72 (64–79)	67 (60 -80)	0.11
Severity Score at Admission_median (IQR)				
SOFA	10 (8–13)	12 (9–14)	9 (7–12)	< 0.0001
SAPS II	65 (53.75–79.25)	73 (63–86)	59 (46–69)	0.002
Charlson	2 (1–4)	3 (2- 5)	1 (0 -3)	< 0.0001
Exposure to Invasive device (Day_ median (IQR)				
Tracheal Intubation	3 (1 -9)	3 (1–9.5)	3 (0–7)	0.16
Venous Catheter	6 (3–13)	4 (2- 12)	7(5–17)	0.002
Urinary Catheter	6 (2–11.7)	4 (1–9)	6 (4–13)	0.006
Primary site of infection, *n* (%)				
Respiratory	31 (20.5)	22 (32.4)	9 (10.8)	0.019
Intra abdominal	69 (46)	26 (38.2)	43 (51.8)	0.009
Urinary	15 (10)	3 (4.4)	12 (14.4)	0.020
Diagnosis Microbiologicaly confirmed, *n* (%)	108 (71.5)	45(66.2)	63 (75.9)	0.083
Nosocomial Infection, *n* (%)	26 (17.2)	9 (13)	17 (20)	0.12
ICU length of Stay	7 (4 -16)	4.5 (2–13)	9 (6 -20)	< 0.0001

**Fig. 1:**
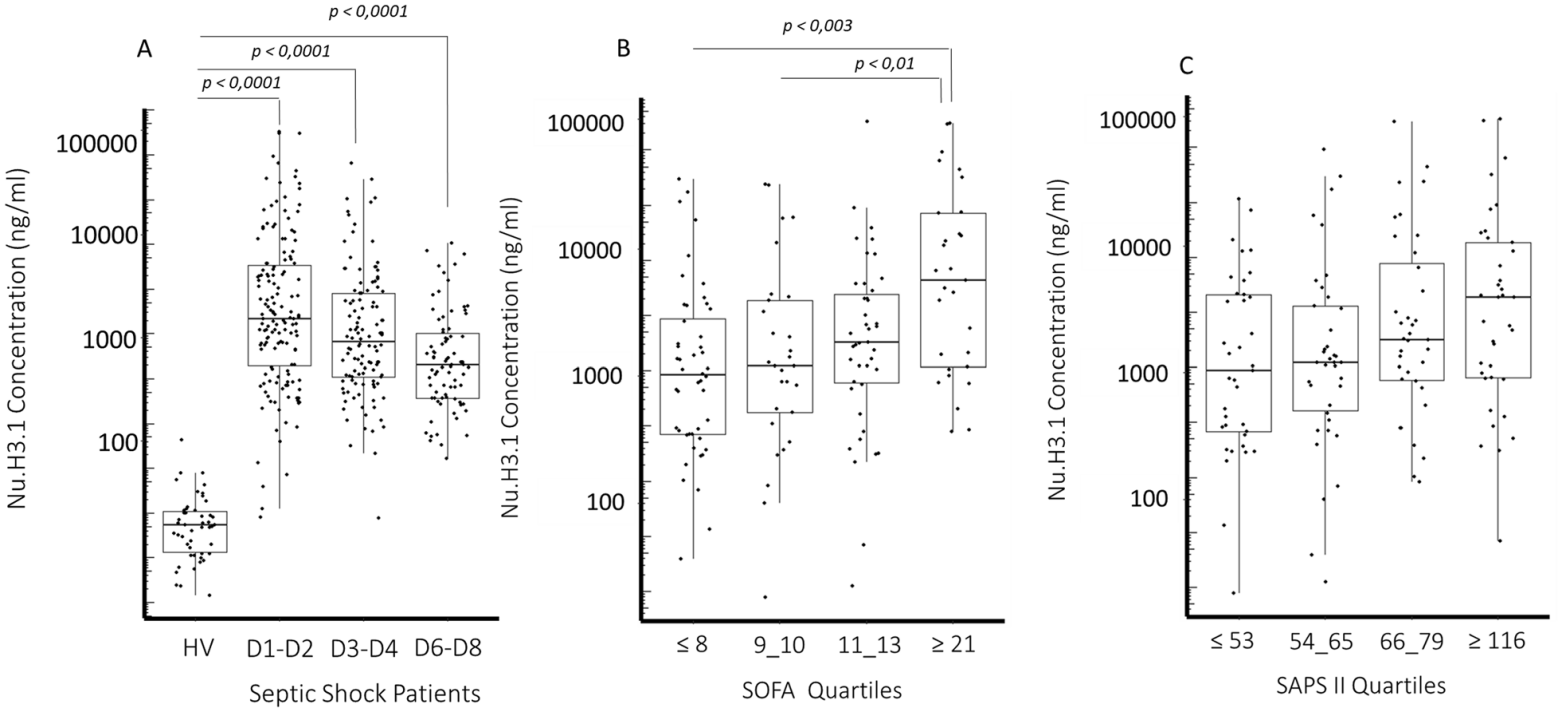
Level of circulating nucleosome H3.1 in septic shock patients. Results are shown as box representing 25th-75th percentile with median and individual values. **A** Nucleosome concentration in healthy volunteers (*n* = 50) and in patients at D1-2 (*n* = 151), D 3–4 (*n* = 116) and D 6–8 (*n* = 78). **B** Association of nucleosome concentration with organ failure assessment score (SOFA) and **C** simplified acute physiology score. This figure represents the nucleosome concentration in patients stratified according to SOFA and SAPS II quartiles upon ICU admission. The Mann–Whitney U test and the Dunn post-hoc test were used for group and more than two group’s comparison

**Fig. 2:**
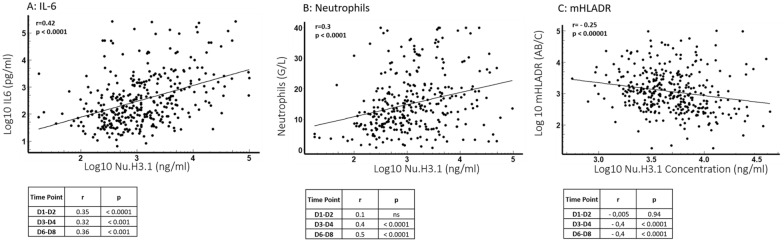
Correlation of nucleosome H3.1 levels with immunological parameters. **A** Correlation with IL-6 (345 samples). **B** Correlation with neutrophil count (314 samples). **C** Correlation with mHLA-DR (Spearman correlation test). Figures depict correlations including all samples plots. Below each figure (ABC), tables are presented, providing Spearman's correlation coefficient and p-value calculated at the different time points for the considered parameter

In this cohort, during the whole monitoring (Fig. 1A), we observed significant and massive elevation of nucleosome H3.1 in comparison with healthy donors (median [15.4 ng/ml]). Levels peaked at Day 1–2 (1515 ng/ml) and then declined progressively. Nevertheless, at Day 6–8, they were still largely above control values. Nu H3.1 concentrations were associated with severity and SOFA scores (Fig. 1B, C). Correlation was found weakly positive but significant with SOFA score (*r* = 0.4, *p* < 0.0001) and SAPS II score (*r* = 0.2, *p* = 0.008). We observed significant positive correlation with inflammatory markers and neutrophil compounds with nucleosome H3.1 (Fig. 2). Regarding IL-6, this correlation was stable over time whereas regarding neutrophils and mHLA-DR it improved with time (Fig. 2). No correlation was observed with lymphocytes. The graphical representation of the results at each point and those depicting correlations in survivors/non survivors are presented in Additional file 1: Figures S1–S4.

**Fig. 3:**
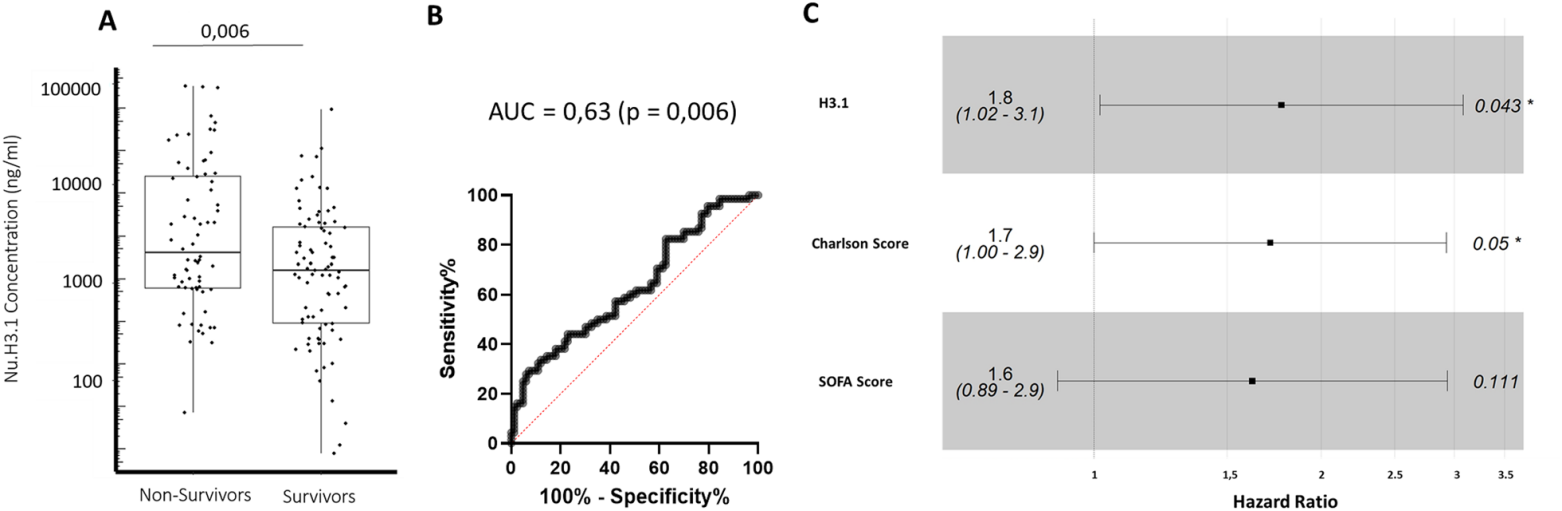
Admission nucleosome H3.1 association with 28-day mortality. **A** Admission level of circulating nucleosomes (day 1–2) in septic shock patients who survived or not. Results are shown as box representing 25th-75th percentile with median and individual values. The Mann–Whitney U test was used for group comparison. **B** Receiver operating characteristic (ROC) curve of admission nucleosomes (day 1–2) for 28-day mortality. AUC = area under the ROC. **C** Multivariate analysis (cox proportional hazard model) at admission (day 1–2) for association with mortality. The patients were divided into two groups based on the highest quartile versus the other quartiles for Nu H3.1, Charlson score, and SOFA score

**Fig. 4:**
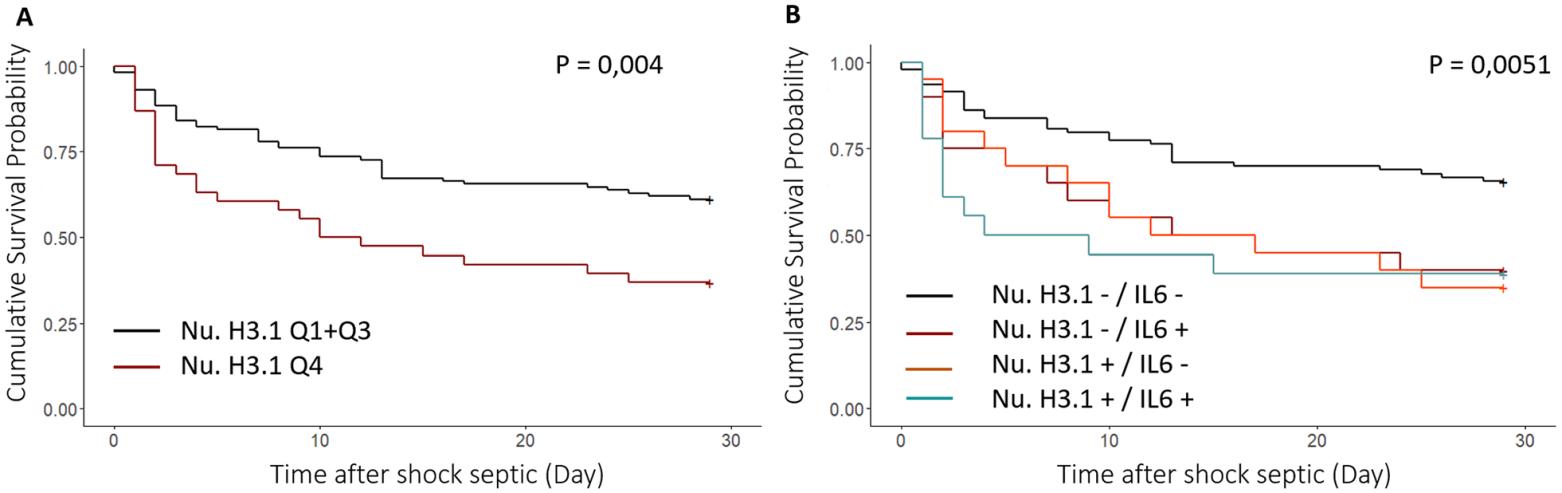
Cumulative incidence of mortality up to 28 day. **A** Patients were stratified into two groups based on the highest quartile (Q4) versus the other quartiles (Q1 + Q3) for Nu H3.1. **B** Patients were stratified into 4 groups based on quartiles: Q4 (labeled +) vs Q1 + Q3 (labeled-) for Nu H3.1 and interleukin-6. First group: Nu H3.1–/ IL-6 – (*n* = 80 patients), second group: Nu H3.1–/IL-6 + (*n* = 20) third group Nu H3.1–/IL-6 + (*n* = 20), fourth group Nu H3.1 + / IL-6 + (*n* = 18). Cumulative incidence curves were estimated with Kaplan–Meier method on D 1–2. The p value was calculated by log rank test. Cumulative incidence curves for day 3–4 and 6–8 Nu H3.1 concentration are shown in Additional file 1: Figure S1

We next examined nucleosome H3.1 results according to 28-day mortality (groups characteristics in Table 1). At Day 1–2 and 3–4, we observed higher values in non-survivor patients (Table 2, Fig. 3A). More precisely, at Day 1–2 (Fig. 3B), area under curve to predict mortality was 0.63 (ROC analysis, *p* = 0.006). Optimal cut-off point (i.e., point closest to the top-left corner), calculated at 4639 ng/mL, was similar to third quartile (5061 ng/mL). Therefore, we next focused on highest values above third quartile and they were found to be independently associated to mortality in multivariate analysis (including Charlson score and SOFA, Fig. 3C). Kaplan–Meier analysis confirmed that elevated Day 1–2 nucleosome H3.1 values were associated with a lower probability of survival (Fig. 4A). This was observed during the whole monitoring (Additional file 1: Figure S5). Meanwhile, non-survivors presented significantly higher IL-6 values at each time-point (Table 2). However, this was not significant after multivariate analysis. Of note, when analyzing both nucleosomes and IL-6 in Kaplan–Meier analysis (both stratified on high Day 1–2 quartile values), we obtained significant values showing that when both markers are elevated, it induced early mortality (Fig. 4B). Considering this later result and the high mortality of the present cohort (45%), we looked at death occurrence over time (Additional file 1: Figure S6) and observed there was an important early mortality, i.e., 53% of total mortality at day 5.

**Table 2:** Nucleosome H3.1 values and Immunological parameters variations according to 28-day mortality

Marker	Time points	Survivor	Non-survivor	*p*
Nu H3.1 (ng/ml)	D1/2	1333,14 (385.14–3637.92)	1919 (880.75–12,098.9)	0.006
	D3/4	662 (378.59–2151.88)	1391.5 (655.8–4186.9)	0.01
	D6/8	530.72 (225.9–960.4)	605.9 (429.3–1851.7)	0.12
IL-6 (pg/ml)	D1/2	745 (245–2849.50)	1859.5 (611.5–19,198.5)	0.004
	D3/4	96 (40–178)	259 (86.25–484.5)	0.0002
	D6/8	54.9 (31.5–106.58)	100 35.92–300.75)	0.09
mHLA-DR (ABC)	D1/2	3793 (2682–6662)	5104 (3136–8374)	0.04
	D3/4	4093 (3128–7172)	3731 (2320–5924)	0.15
	D6/8	7252 (4793–9939)	4762 (2700–6391)	0.009
Lymphocyte (G/L)	D1/2	0.905 (0.500–1.600)	1.05 (0.61–1.56)	0.40
	D3/4	0.9 (0.600–1.215)	0.73 (0.500–1.200)	0.53
	D6/8	1.03 (0.900–1.200)	1.100 (0.800–1.600)	0.76

Results at different time points as median and (interquartile range). *p* Mann–Whitney test

**Fig. 5:**
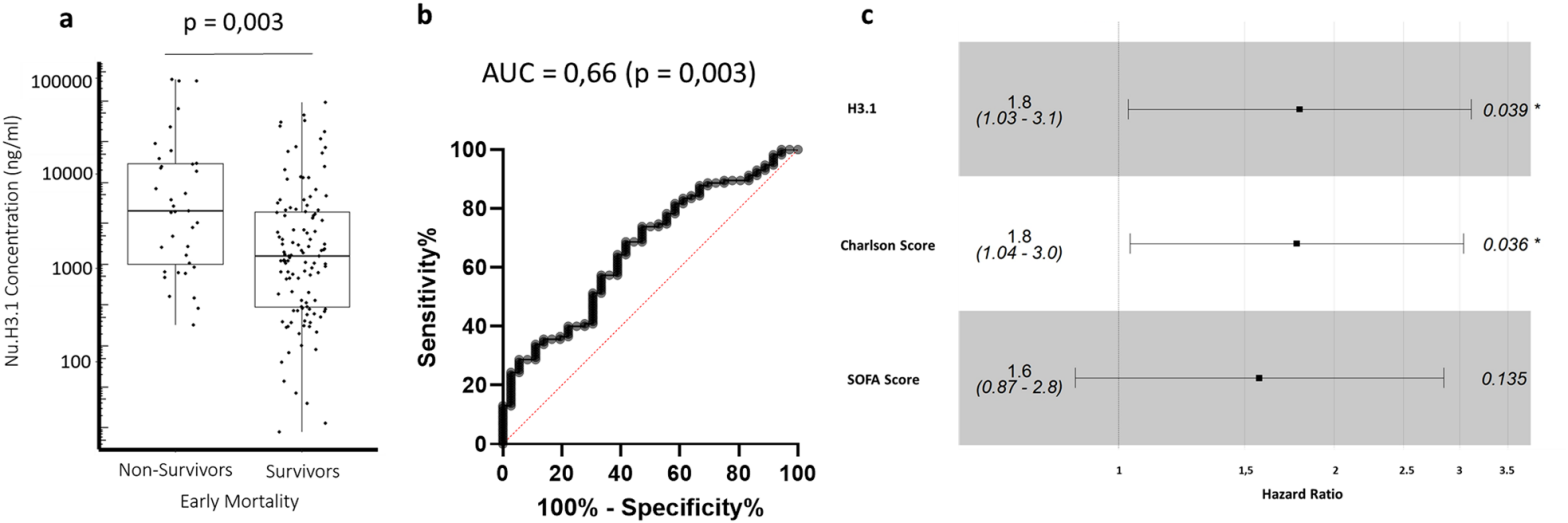
Admission (day 1–2) nucleosome H3.1 association with 5-day mortality. **A** Admission level of circulating nucleosomes (day 1–2) in septic shock patients who survived or not at day-5. Results are shown as box representing 25th–75th percentile with median and individual values. The Mann–Whitney U test was used for group comparison. **B** Receiver operating characteristic (ROC) curve of admission nucleosomes (day 1–2) for 5-day mortality. AUC = area under the ROC **C** Multivariate analysis (cox proportional hazard model) at admission (day 1–2) for association with 5-day mortality. The patients were divided into two groups based on the highest quartile versus the other quartiles for Nu H3.1, Charlson score, and SOFA score

Thus, we investigated whether Day 1–2 high nucleosome H3.1 values (i.e., upper quartile) were associated with day-5 mortality (Fig. 5). In this case, area under curve was improved (0.66, *p* = 0.003) and highest values remained independently associated to mortality (Fig. 5C). It was not the case for IL-6.

## Discussion

The advent of the standardized NuQ H3.1® assay for automated and standardized quantification of nucleosomes has opened up new avenues for further exploration and research in this field. To our knowledge, we report the first study that explores nucleosomes H3.1 in a substantial cohort of septic shock patients who simultaneously exhibit a severe inflammatory response and profound alterations of immune cellular parameters. With the present study, we provide several important results.

The first one is to demonstrate that circulating nucleosome H3.1 level was markedly increased in patients upon admission. In addition, and as expected, we found positive correlation between nucleosome H3.1 concentrations, interleukin 6, a pro-inflammatory cytokine, and neutrophils the responsible cells for NETs formation. Interestingly, we observed an increased correlation over time with neutrophils, suggesting that after initial tissue aggression, which could generate intranuclear material by different mechanisms, the production of nucleosomes is increasingly dependent on residual activation of neutrophils. This point would require additional investigations to further explore this finding.

The second one is to show that higher nucleosome concentrations were significantly associated with SOFA and severity scores and, most importantly, with both 5- and 28-day mortality in an independent manner. Indeed, when we focused on the upper quartile of nucleosome values, this association remained significant in multivariate analysis, indicating that nucleosome levels alone can provide valuable clinical information. In contrast, significant differences in interleukin-6 (IL-6) levels between survivors and non-survivors disappeared after multivariate analysis. However, Kaplan–Meier analysis demonstrated that, when associated, higher values of both nucleosome and IL-6 at admission identified a subset of patients who died very rapidly. Therefore, nucleosomes may contribute to the establishment of a hyper-inflammatory phenotype (along with other inflammatory markers), which could guide clinicians toward very aggressive anti-inflammatory strategies, such as personalized corticosteroid use.

A third important finding was the persistent high nucleosome values observed over time. Even at the end of the first week, these values remained largely above the control values. Of particular significance was the association between the Day 6–8 nucleosome values and 28-day mortality (Additional file 1: Figure S1). Given that higher nucleosome values were linked to lower Day 6–8 mHLA-DR (Fig. 2), the question arises as to whether nucleosome and persistence of inflammation contribute to delayed immunosuppression. The clearance mechanisms for NETs are not completely understood. In experimental infection models, NETs have been observed to persist for several days and are believed to be broken down by plasma nuclease DNAse I. However, even after DNA degradation, NETs can still persist, indicating the involvement of additional clearance mechanisms. One possible mechanism could be the scavenging properties of monocytes which are known to be impaired in sepsis [31]. Further research is needed to explore the potential causal relationship between nucleosomes and low mHLA-DR levels.

Overall, the current findings support previous research indicating that various compounds released during NET formation, such as histones, circulating cell-free DNA, and myeloperoxidase, are associated with mortality or negative outcomes in septic patients [32–35]. However, none of these compounds, including nucleosomes, are, by themselves, specific to NETs and can also be released in response to other tissue damage. Thus, numerous mediators have the potential to serve as biomarkers of NET formation. Therefore, the analytical aspects of measuring these compounds, particularly in clinical research, need to be considered. Currently, ELISA assays and home-made protocols are the main methods for measuring NET products. As such, these tests present several analytical challenges to clinical deployment, such as manual performance, preparation procedures, the need for skilled technicians, limited access, and poor standardization. As a result, the poor reproducibility and reliability of these assays pose significant challenges to their practical use in clinical settings. Therefore, an automated NET assay may provide a more standardized and reliable approach to investigating NET formation in clinical settings.

Beyond its interest as biomarker, the involvement of NETs and related products in various inflammatory and immune-mediated diseases highlights the potential of targeting these pathways for the development of novel therapeutic strategies. In this context, elevated nucleosomes may indicate a treatable trait. Clinical development of inhibitors to directly target NET formation, has already been started [23, 30, 36, 37]. In addition, therapeutic plasmapheresis (i.e., the selective extra corporeal removal of NET) is under investigation (NCT04749238). In case of favorable preliminary results, nuclesosome levels would constitute an obvious candidate to guide individualized therapy as companion biomarker.

This study has some limitations that need to be addressed. Firstly, the cohort used in the study consisted of critically ill septic shock patients with a high mortality rate. Therefore, the results of the study cannot be generalized to all septic patients. Secondly, this study was conducted retrospectively using the IMMUNOSEPSIS cohort, which was primarily aimed at monitoring immunosuppression (NCT02803346). Thus, a prospective study, including longer follow-up, should be designed to specifically explore nucleosome, along with other relevant markers, to obtain more in-depth information regarding its potential as a biomarker and/or as an actor of delayed immune deregulation in sepsis.

## Conclusion

This first study based on standardized nucleosome measurement sets important milestones in the field of sepsis. Over a 1 week follow-up period allowing dynamic changes, it demonstrates a significant increase in circulating nucleosomes that was associated with early mortality and 28-day mortality in a substantial cohort of patients with septic shock. This elevation was also associated with neutrophil count, IL-6 concentration, and decreased mHLA-DR. Upon confirmation, nucleosome measurement could become a preferred marker in routine clinical practice.

### Supplementary Information

**Additional file 1: Figure S1.** Correlation of nucleosome H3.1 levels with immunological parameters. **Figure S2.** Correlation of nucleosome H3.1 levels with IL_6. **Figure S3.** Correlation of nucleosome H3.1 levels with Neutrophils. **Figure S4.** Correlation of nucleosome H3.1 levels with mHLADR. **Figure S5.** Cumulative incidence of mortality up to one-month (day 28). **Figure S6.** Histogram of distribution of number of deaths per day after ICU admission.

**Additional file 2: Table S1.** Immunological results at different time points during the first week after ICU admission.

## Data Availability

The original contributions presented in the study are included in the article. Further inquiries can be directed to the corresponding author.

## Abbreviations

AUC: Area under curve
COVID-19: Coronavirus Disease
DAMPS: Damage-associated molecular patterns
DNA: Deoxyribonucleic acid
EDTA: Ethylenediamine tetraacetic acid
ELISA: Enzyme linked immunoassay test
ICU: Intensive care unit
IL-6: Interleukin-6
mHLADR: Monocytic HLADR
NETs: Neutrophils extracellular traps
NU. H3.1: Nucleosome containing histone 3.1
RLU: Relative Light Unit
ROC: Receiver operating characteristic curve
SAPS II: Simplified acute physiology score
SOFA: Sequential organ failure assessment
