# Supplementary material for: Association of pronounced elevation of NET formation and nucleosome biomarkers with mortality in patients with septic shock
Source: Ann Intensive Care. 2023 Oct 17;13:102. doi: 10.1186/s13613-023-01204-y (PMC10581968; doi:10.1186/s13613-023-01204-y)
Supplement: Supplementary file 1 — Additional file 1: Figure S1. Correlation of nucleosome H3.1 levels with immunological parameters. Figure S2. Correlation of nucleosome H3.1 levels with IL_6. Figure S3. Correlation of nucleosome H3.1 levels with Neutrophils. Figure S4. Correlation of nucleosome H3.1 levels with mHLADR. Figure S5. Cumulative incidence of mortality up to one-month (day 28). Figure S6. Histogram of distribution of number of deaths per day after ICU admission. [file 13613_2023_1204_MOESM1_ESM.pdf]

Association of pronounced Elevation of NET formation and Nucleosome Biomarkers with Mortality in Patients with Septic Shock

Additional

Additional FIGURES

Figure S1

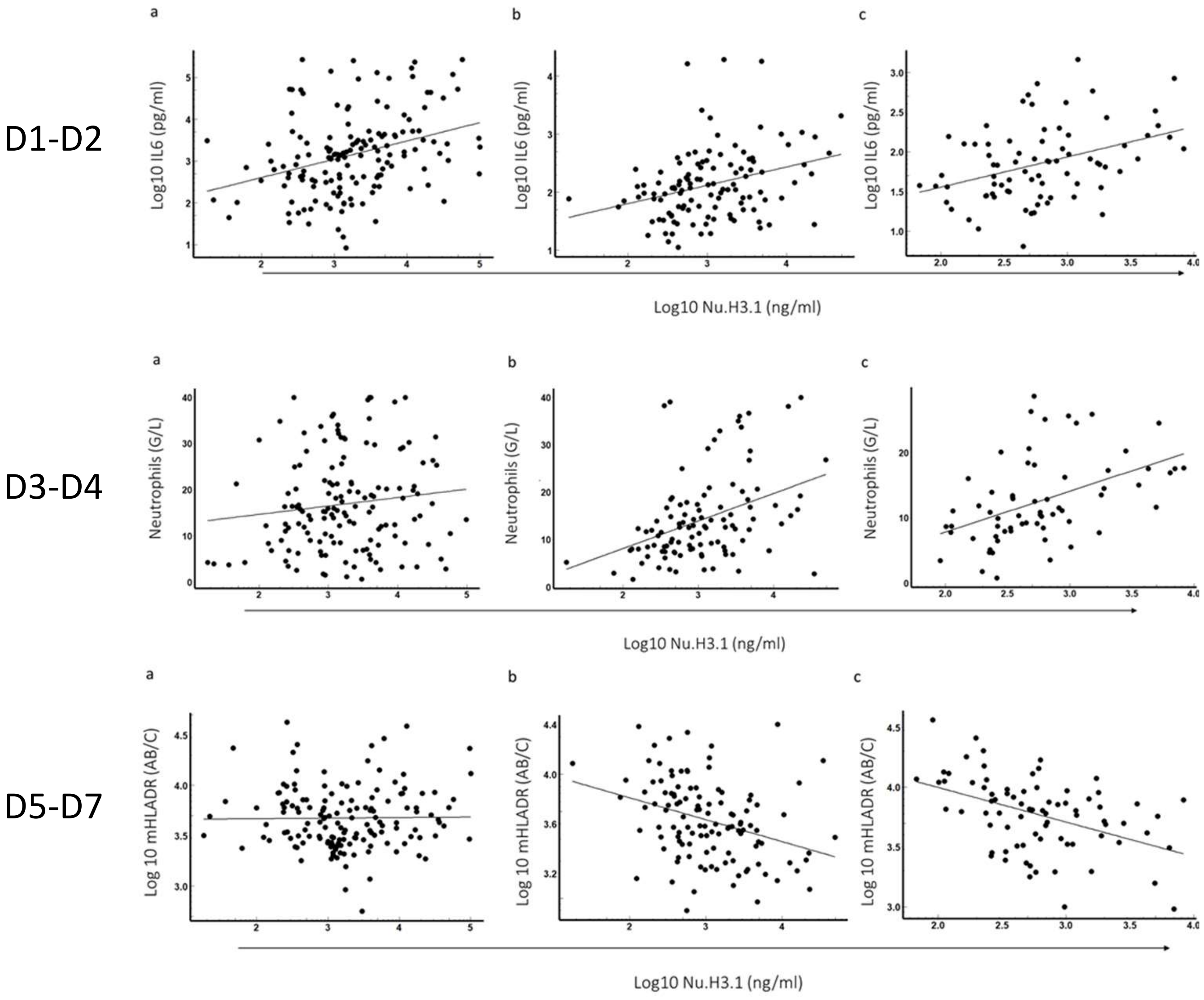

	IL-6		Neutrophils		mHLADR	
Time Points	r	P	r	P	r	P
(a) D1-D2	0.35	< 0,0001	0.1	ns	-0,005	0,94
(b) D3-D4	0.32	< 0,001	0.41	< 0,001	-0,4	< 0,0001
(c) D6-D8	0.36	< 0,001	0.5	< 0,0001	-0,4	< 0,0001

Additional file 1 Figure S1. Correlation of nucleosome H3.1 levels with immunological parameters. Figures depict correlations at different time points a (D1-D2), b (D3-D4), c (D6-D8). Tables presented, providing Spearman's correlation coefficient and p-value calculated at different time points for the considered parameter.

Additional file 1:

Figure S2

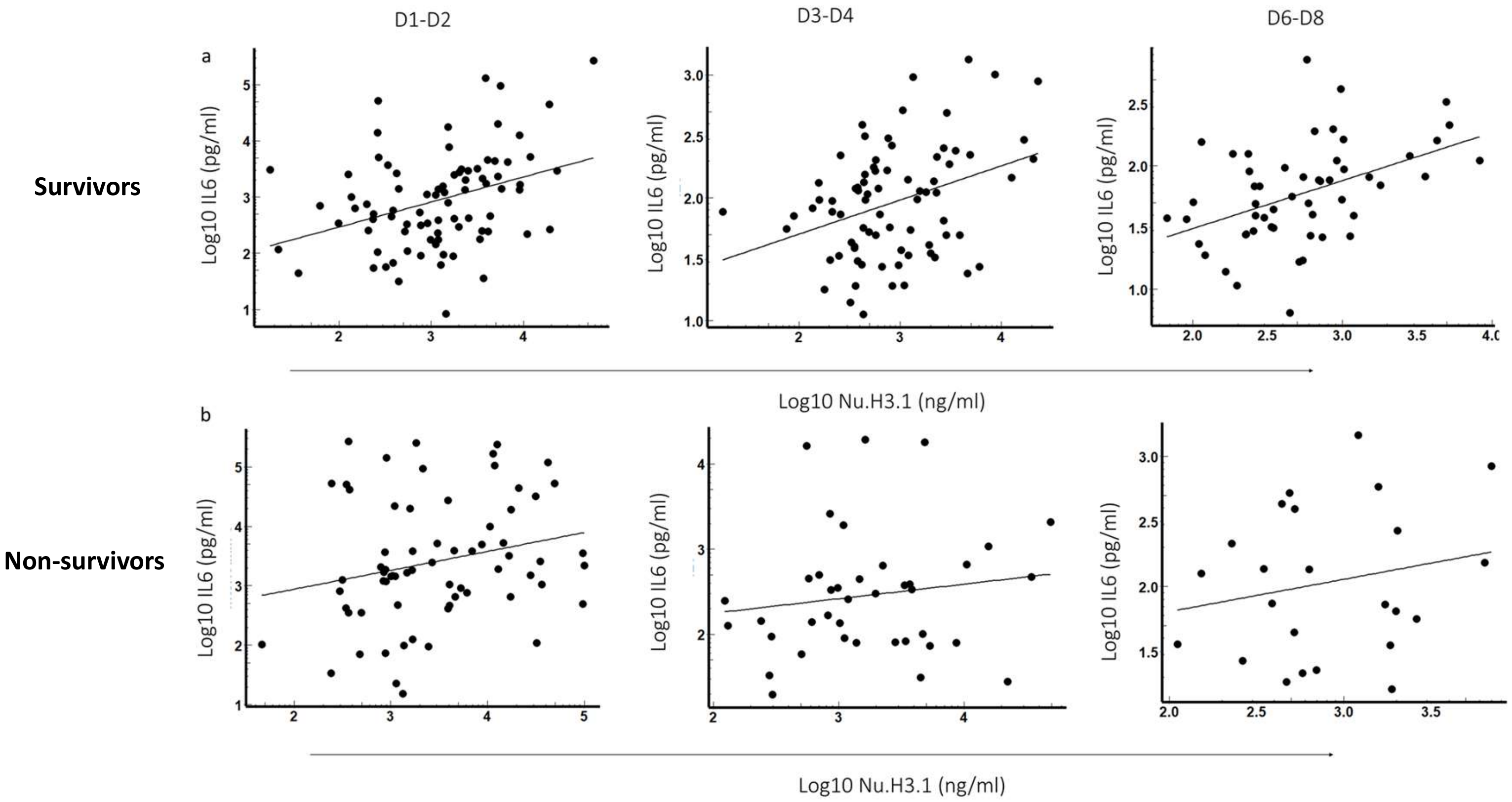

Time Points	a (Survivor)		b (No Survivor)	
	r	P	r	P
D1-D2	0,34	0,001	0,25	0,039
D3-D4	0,3	0,006	0,15	0,35
D6-D8	0,4	0,0003	0,1	0,5

Additional file 1 Figure S2. Correlation of nucleosome H3.1 levels with IL_6. Figures depict correlations at different time points a; for survivors and b; for no survivors. Tables presented, providing Spearman's correlation coefficient and p-value calculated at different time points respectively from top to bottom.

Additional file 1:

Figure S3

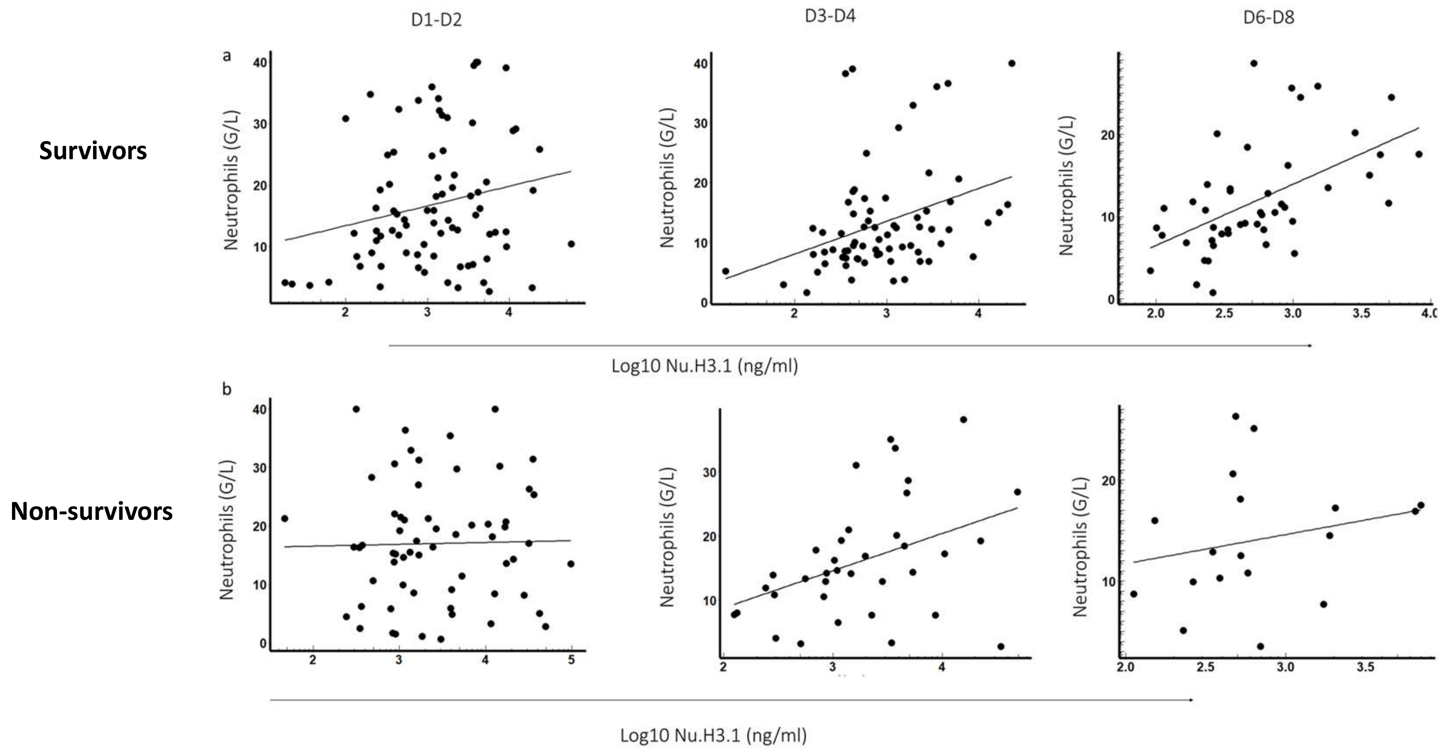

Time Points	a (Survivor)		b (No Survivor)	
	r	P	r	P
D1-D2	0,17	0,1	0,04	0,1
D3-D4	0,38	0,0009	0,43	0,008
D6-D8	0,59	<0.0001	0,26	0,28

Additional file 1 Figure S3. Correlation of nucleosome H3.1 levels with Neutrophils. Figures depict correlations at different time points a; for survivors and b; for no survivors. Tables presented, providing Spearman's correlation coefficient and p-value calculated at different time points respectively from top to bottom.

Additional file 1:

Figure S4

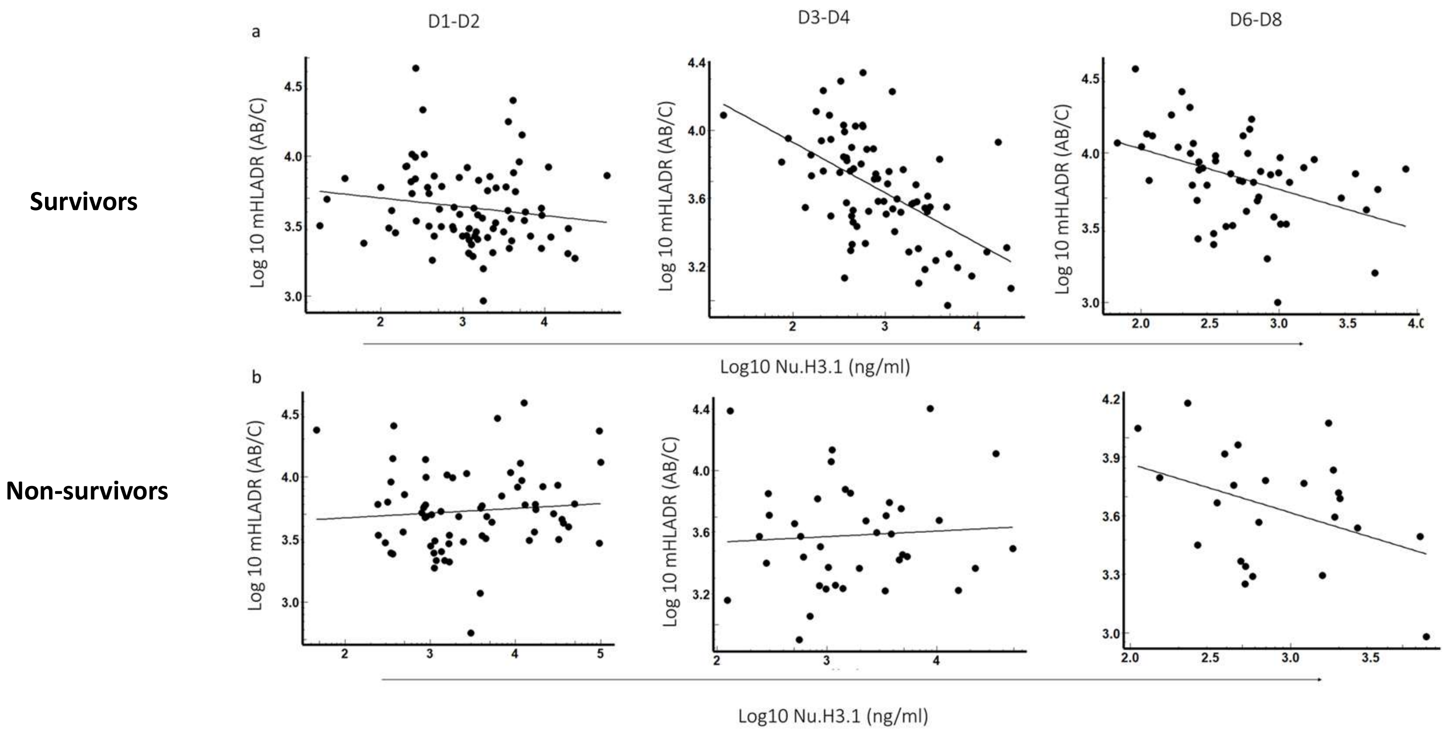

Time Points	a (Survivor)		b (No Survivor)	
	r	P	r	P
D1-D2	-0,18	0,098	0.48	0,087
D3-D4	- 0.5	< 0.0001	0.5	0,098
D6-D8	-0,4	0,0006	-0,34	0,09

Additional file 1 Figure S4. Correlation of nucleosome H3.1 levels with mHLADR. Figures depict correlations at different time points a; for survivors and b; for no survivors. Tables presented, providing Spearman's correlation coefficient and p-value calculated at different time points respectively from top to bottom.

Additional file 1:

Figure S5

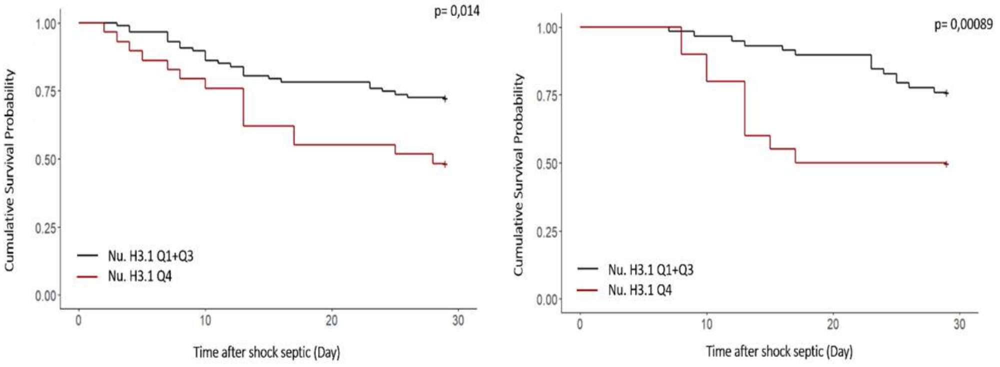

additionalfile Figure S5. Cumulative incidence of mortality up to one-month (Day 28). Patients were stratified to two groups based on Nu.H3.1 quartiles. Cumulative incidence curves of (A) D3-D4 and (B) D6-D8 based on Nu.H3.1 concentration. Cumulative incidence curves were estimated with Kaplan-Meier method. The p value was calculated by log rank test.

Additional file 1:

Figure S6

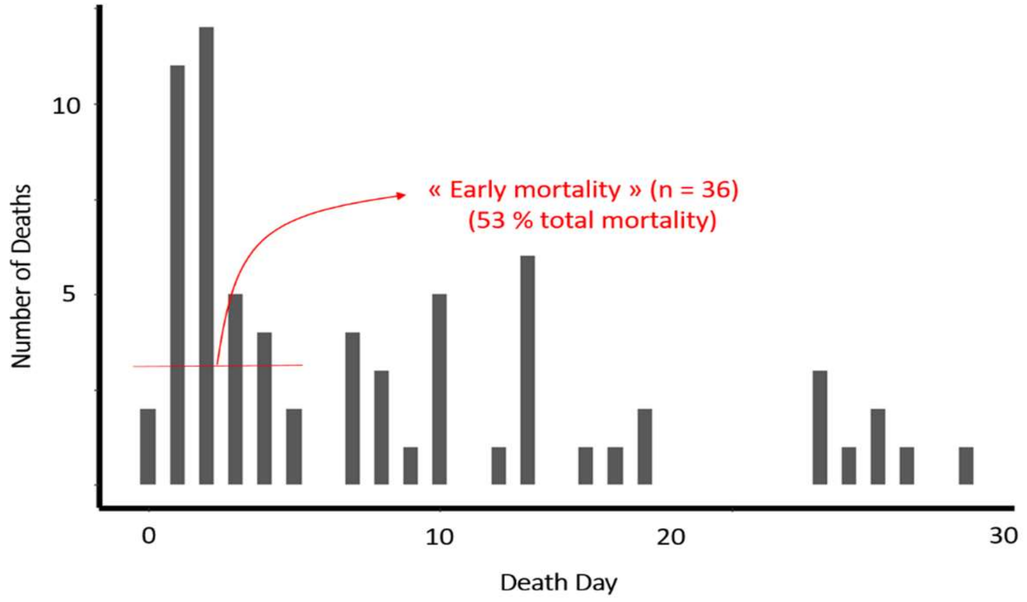

Additional file 1 Figure S6. Histogram of distribution of number of deaths according to deaths days.

Additional file 1:
