# Supplementary material for: Association of pronounced elevation of NET formation and nucleosome biomarkers with mortality in patients with septic shock
Source: Ann Intensive Care. 2023 Oct 17;13:102. doi: 10.1186/s13613-023-01204-y (PMC10581968; doi:10.1186/s13613-023-01204-y)
Supplement: Supplementary file 2 — Additional file 2: Table S1. Immunological results at different time points during the first week after ICU admission. [file 13613_2023_1204_MOESM2_ESM.pdf]

Association of pronounced Elevation of NET formation and Nucleosome Biomarkers with Mortality in Patients with Septic Shock

Additional file 2: Table S2

Muzhda Haem Rahimi et al.

Additional file 2 Table S2 . Immunological results at different time points during the first week after ICU admission.

Parametres	D 1-2	D 3-4	D 6-8	Reference values
IL-6 (pg/ml)	1335 (338 - 3872)	114 (52 - 276)	68 (32 - 133)	< 7
mHLA-DR (AB/C)	4295 (2926 - 7011)	3853 (2526 - 6919)	6374 (3703 - 9298)	15500 - 45000
Leukocytes (G/L)	17.30 (9.7 - 26.5)	16.7 (9.4 - 21)	12.9 (10.3 - 19.2)	4 -10
Neutrophils (G/L)	15.3 (8.6 - 21.8)	12.5 (8 - 17.4)	11 (8.4 - 16.9)	2 - 7.5
Lymphocytes (G/L)	0.97 (0.5 -1.6)	0.8 (0.5 - 1.2)	1.04 (0.83 - 1.35)	1 - 2.8
CD4 (Cells/µl)	na	338 (221 - 541)	na	336 - 1126

Reference values are provided by routine clinical immunology laboratories at our institution. Results as median and (interquartile range). Na = not available.
